# ROS Induced by KillerRed Targeting Mitochondria (mtKR) Enhances Apoptosis Caused by Radiation via Cyt c/Caspase-3 Pathway

**DOI:** 10.1155/2019/4528616

**Published:** 2019-03-07

**Authors:** Xin Li, Fang Fang, Ying Gao, Geng Tang, Weiqiang Xu, Yihan Wang, Ruoxian Kong, Ayixianguli Tuyihong, Zhicheng Wang

**Affiliations:** NHC Key Laboratory of Radiobiology, School of Public Health, Jilin University, Changchun 130021, China

## Abstract

During radiotherapy, reactive oxygen species- (ROS-) induced apoptosis is one of the main mechanism of radiation. Based on KillerRed which can induce ROS burst in different cell substructures, here we hypothesized that KillerRed targeting mitochondria (mtKR) could induce ROS to enhance apoptosis by radiation. In this study, empty vector, mtKR, and mtmCherry plasmids were successfully constructed, and mitochondrial localization were detected in COS-7 and HeLa cells. After HeLa cells were transfected and irradiated by visible light and X-rays, ROS levels, mitochondrial membrane potential (Δ*ψ*_m_), ATPase activities, adenosine triphosphate (ATP) content, apoptosis, and the expressions of mRNA and protein were measured, respectively. Data demonstrated that the ROS levels significantly increased after light exposure, and adding extra radiation, voltage-dependent anion channel 1 (VDAC1) protein increased in the mitochondria, while Na^+^-K^+^ and Ca^2+^-Mg^2+^ ATPase activities, ATP content, and Δ*ψ*_m_ significantly reduced. Additionally, the cell apoptotic rates dramatically increased, which referred to the increase of cytochrome c (Cyt c), caspase-9, and caspase-3 mRNA expressions, and Cyt c protein was released from the mitochondria into the cytoplasm; caspase-9 and -3 were activated. These results indicated that mtKR can increase the production of ROS, enhance mitochondrial dysfunction, and strengthen apoptosis by radiation via Cyt c/caspase-3 pathway.

## 1. Introduction

Mitochondria are essential organelles for cell survival, death, and signaling and are also one of the main production sites of reactive oxygen species (ROS) [1, 2]. In addition, mitochondria also play a prominent role in the regulation of apoptosis [3–5]. When ROS is produced in the mitochondria, adenosine triphosphate (ATP) is also produced. ROS can be generated endogenously during cellular respiration or in response to infection and can be induced exogenously by chemical and physical agents, such as radiation, UV, and cigarette smoke. Lower levels of ROS play a role in normal cellular function [6], while increased levels of ROS induce oxidative stress which is the cause or consequence of the damage to mitochondria and mitochondrial DNA (mtDNA) [7].

In addition, mitochondria are also a damaging target of ROS. Under normal physiological conditions, ROS resulting from mitochondria is removed by a cellular antioxidant defense system. However, once ROS is overproduced, it will lead to the accumulation of excess radicals that damage the mitochondria and cells [8]. The literatures suggest that oxidative damage has also played a key role in diseases such as diabetes, Parkinson's disease, Alzheimer's disease, and even in the progress of cancers [9–11]. And ROS may mediate the programmed cell death (PCD) at a moderately high concentration among different cell types [12, 13]. In apoptosis, external stimuli such as radiation and cytotoxic agents can result in the formation of pores at mitochondrial membranes. Disruption of mitochondrial membrane potential (Δ*ψ*_m_) is a major sign of mitochondrial dysfunction. Loss of the Δ*ψ*_m_ can result in a defective mitochondrial electron transport chain (ETC) and decrease metabolic oxygen consumption and ATP depletion [14]. Mitochondrial dysfunction results in the release of proapoptotic protein Cyt c and activates caspases to induce apoptosis. Once mitochondrial permeability transition pore (MPTP) is activated by oxidative stress, the membrane depolarization will develop, and the uncoupling of oxidative phosphorylation and ATP depletion will be induced [15]. Nowadays, the strategy targeting mitochondrial dysfunction in cancer therapy has been the research hotspots [16].

Radiotherapy is a conventional mean for cancer treatment for several decades. There is a growing interest in understanding how the altered mitochondrial functions may be the target to improve the effects of radiotherapy [17]. The modified bioenergetic and biosynthetic states of mitochondria play an eminent role for cancer cells in response to radiation [18]. Radiotherapy causes death of cancer cells through apoptosis and autophagy induced by excessive production of ROS [19–21]. Therefore, how to induce enough ROS to target mitochondria is a crucial research topic. KillerRed can directly express in cells, and under appropriate light excitation, it can efficiently induce ROS to cause cell death [22–25]. KillerRed can be used for the inactivation of light-induced protein, killing specific cell populations *in vivo* and studying intracellular local oxidative stress [26–28]. Additionally, because of light-inducing inactivation of KillerRed, in some studies, KillerRed was replaced by another red fluorescence protein mCherry (no phototoxicity) to study intracellular localization.

In this study, the N-terminal mitochondrial-targeting sequence (MTS) of PTEN-induced putative kinase 1 (Pink1) was used to mediate downstream mCherry and KillerRed to express in mitochondria [29]. Under fluorescence microscope, the colocalization of mCherry (red) and mitochondrial tracker COX IV (green) in both African green monkey kidney cell COS-7 and human cervical cancer cell HeLa was observed. Furthermore, we explored mtKR-induced mitochondrial dysfunction and apoptosis by light and X-rays, and proapoptotic mechanisms via Cyt c/caspase-3 pathway, to provide a new idea for cancer radiotherapy.

## 2. Materials and Methods

### 2.1. The mtmCherry and mtKR Vectors

**Figure 1:**
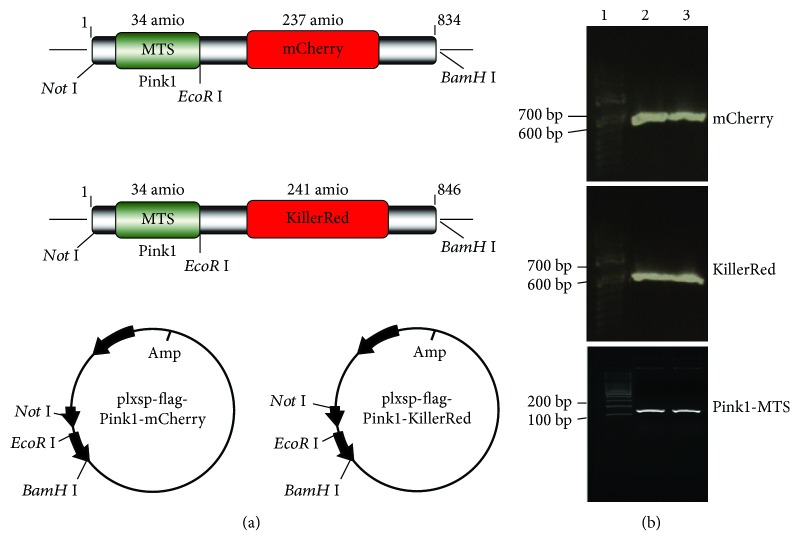
Development of vectors for mtmCherry and mtKR. (a) Schematic diagram of mtmCherry and mtKR vectors: Pink1-MTS was cloned into empty vector (plxsp-flag) (*Not* I and *EcoR* I sites); mCherry and KillerRed were cloned into plxsp-flag-Pink1-MTS (*EcoR* I and *BamH* I sites). (b) PCR products of mCherry, KillerRed, and Pink1-MTS. Lane 1 was 100 bp DNA Marker; lane 2 and 3 were PCR amplification products.

In this study, the DNAs of mCherry, KillerRed, and Pink1-MTS were amplified with PCR using Q5 High-Fidelity DNA Polymerase (NEB, Beverly, MA, USA), and plxsp-TetA-mCherry, plxsp-TetA-KillerRed (kindly given by Dr. Shen from Cancer Institute of New Jersey, USA), and pcDNA-DEST47 PINK1 C-GF plasmids (Addgene, Cambridge, MA, USA) were used as templates. The following primers were used: mCherry: 5´-GGAATTCGCCACCATGGTGAGCAAGGG-3´(F), 5´-CGGGATCCTTACTTGTACAGCTCGTCCATG-3´(R); KillerRed: 5´-GGAATTCATGGGTTCAGAGGGC-3´(F), 5´-CGGGATCCCTAGATCTCGTCG-3´(R); Pink1-MTS: 5´-AAGGAAAAAAGCGGCCGCAATGGCGGTGCGACAG-3´(F), 5´-CGAATTCCGGCCGCCCCAAGCCGTAG-3´(R). The schematic diagram was shown in Figure 1(a), and the PCR products of mCherry, KillerRed, and Pink1-MTS were shown in Figure 1(b). All of the resulting plasmids were sequenced to verify that the clones had the correct sequence.

### 2.2. Cell Transfection and Observation with Fluorescence Microscope

COS-7 and HeLa cells were obtained from the ATCC (American Type Culture Collection). Both cell lines were maintained at 37°C under humidified conditions and 5% CO_2_ and cultured in Dulbecco's modified Eagle's medium (DMEM, Gibco, Grand Island, NY, USA), supplemented with 10% fetal bovine serum (MRC, Jiangsu, China). COS-7 and HeLa cells were seeded into a 6-well plate with coverslips at 2 × 10^5^/well and routinely incubated for 8-12 h without light. The mtmCherry plasmids were transfected into the cells with Hieff Trans™ Liposomal Transfection Reagent (Shanghai YESEN Biotechnology Co., Ltd.). At 30 h posttransfection, the coverslips were taken out, and the cells were fixed in PBS with 4% paraformaldehyde for 10 min at room temperature (RT), permeabilized and blocked with sealing fluid (0.3% Triton X-100 and 2% BSA in PBS) for 1 h at RT. The cells were incubated with COX IV antibody diluted in sealing fluid overnight at 4°C, followed by incubation with secondary antibodies (green fluorescence) diluted in sealing fluid for 1 h at 37°C. The coverslips were mounted onto microscope slides; mCherry and COX IV expressions were observed under fluorescence microscope. The images were processed for analyzation.

### 2.3. ROS Detection

HeLa cells were transfected with empty vector and mtKR plasmids for 30 h and exposed to visible light for 10, 30, and 60 min, respectively, then at 10, 30, and 60 min after exposure, 2′,7′-dichlorofluorescein diacetate (DCFH-DA, Sigma-Aldrich, St. Louis, MO, USA) was added into the cells. Finally, the mean fluorescence intensity (MFI) was detected by Cytation™ 3 Cell Imaging Multi-Mode Reader System (BioTek, Winooski, Vermont, USA). There were 6 replicate wells per group, and the experiment was performed in triplicate.

### 2.4. Detections of Na^+^-K^+^ and Ca^2+^-Mg^2+^ ATPase Activities and ATP Content

At 12 h postlight exposure, HeLa cells were irradiated by 4 Gy X-rays with X-RAD 320iX machine (Precision X-ray, Inc., USA), at 24 h postirradiation, the cells were homogenized using homogenate medium (pH 7.4, 0.01 M Tris-HCl, 0.001 M EDTA-2Na, 0.01 M saccharose, and 0.8% NaCl) (Nanjing Jiancheng Bioengineering Institute, China), and protein concentrations were determined. Na^+^-K^+^ and Ca^2+^-Mg^2+^ ATPases and ATP were measured using biochemical assay kits (Nanjing Jiancheng Bioengineering Institute, China) and a spectrophotometer (Beckman, USA) with 636 nm excitation wavelengths. There were 4 replicate wells per group, and the experiment was performed in triplicate.

### 2.5. Flow Cytometry (FCM)

Rhodamine123 (Rh123, Sigma-Aldrich, St. Louis, MO, USA) was used to detect Δ*ψ*_m_, and Annexin V-FITC/PI kit (Becton, Dickinson and Company, Franklin Lakes, NJ, USA) was used to measure apoptotic rate. The collected cells were resuspended at 12 h postirradiation, then, Rh123 was added into the cells to yield final concentrations of 5 *μ*M for detecting Δ*ψ*_m_ and stained with 10 *μ*l Annexin V-FITC and PI for 15 min in the dark for detecting apoptotic rate. Then the Δ*ψ*_m_ and apoptotic rate were detected by FCM (Becton, Dickinson and Company, Franklin Lakes, NJ, USA). For each sample, at least 1 × 10^4^ cells were collected. There were 4 replicate wells per group. The experiment was performed in triplicate.

### 2.6. Quantitative Real-Time PCR (qRT-PCR)

Total RNA was extracted with TRIzol reagents (Invitrogen, Carlsbad, CA, USA), and the complementary DNA (cDNA) was synthesized using a high-capacity reverse transcription kit (Takara Bio Inc., Japan). The reverse transcription of 1 *μ*g RNA was performed according to the protocol, and the reaction was incubated at 42°C for 60 min, then at 70°C for 2 min. GAPDH: 5′-ACCACAGTCCATGCCATCAC-3′(F), 5′-TCCACCACCCTGTTGCTGTA-3′(R); Cyt c: 5′-GGGCGAGAGCTATGTAATGCAAG-3′(F), 5′-TACAGCCAAAGCAGCAGCTCA-3′(R); caspase-9: 5′-GGACATCCAGCGGGCAGG-3′ (F), 5′-TCTAAGCAGGAGATGAACAAAGG-3′(R); caspase-3: 5′-TTCAGGCCTGCCGTGGTACA-3′(F), 5′-CCAAGAATAATAACCAGGTGCT-3′(R). The qRT-PCR reaction was performed and analyzed (Bio-Rad, Hercules, CA, USA) according to SYBR® Premix Ex Taq ™ II kit (Takara Bio Inc., Japan) protocol.

### 2.7. Mitochondrial Protein Extraction

The cells were washed with 0.01 M PBS and collected at × 200 g for 5 min, added with 3 ml mitochondrial separation reagents (Beyotime® Biotechnology, Hangzhou, China) consisting of PMSF and put on ice for 10 min. The cell homogenate was transferred into glass homogenizer, performed for 30 min, and centrifuged at × 600 g at 4°C for 10 min. The suspension was transferred to another tube and centrifuged at × 11000 g at 4°C for 10 min. When the suspension was removed after centrifugation, the mitochondria were obtained. Then the mitochondrial proteins were extracted and quantitatively determined.

### 2.8. Western Blot

After the total proteins were extracted and quantitatively determined, 40 *μ*g proteins were separated by SDS-PAGE (10% resolving gel, 5% stacking gel) and transferred to NC membrane (200 mA, 1.5 h; Merck Millipore, Billerica, MA, USA). After blocking with 5% nonfat dry milk, the membranes were incubated with diluting solution (1 : 200) of the primary antibodies including anti-VDAC1, anti-HSP60 and anti-Cyt c (Bioworld Technology Inc., USA), anti-caspase-9 (cleaved) and anti-caspase-3 (cleaved) (Cell Signaling Technology, Danvers, MA, USA), and anti-GAPDH (Santa Cruz, CA, USA), respectively, overnight at 4°C. After washing with TBST, the membranes were incubated with IgG-HRP-conjugated secondary antibody (ImmunoWay, Plano, TX, USA) at 1 : 1000 dilution for 1.5 h at RT. Finally, the membranes were identified using an enhanced chemiluminescence detection system (ECL detection kit, Santa Cruz, CA, USA). The films were scanned for the following gray scale ratio analysis.

### 2.9. Statistical Analysis

All the data were analyzed using SPSS, version 24.0 (SPSS Inc., Chicago, IL, USA). The results were presented as mean ± SD and subjected to one-way ANOVA followed by Student's *t*-test; *P* < 0.05 was considered as significant.

## 3. Results

### 3.1. The mtmCherry Protein to Localize Mitochondria

**Figure 2:**
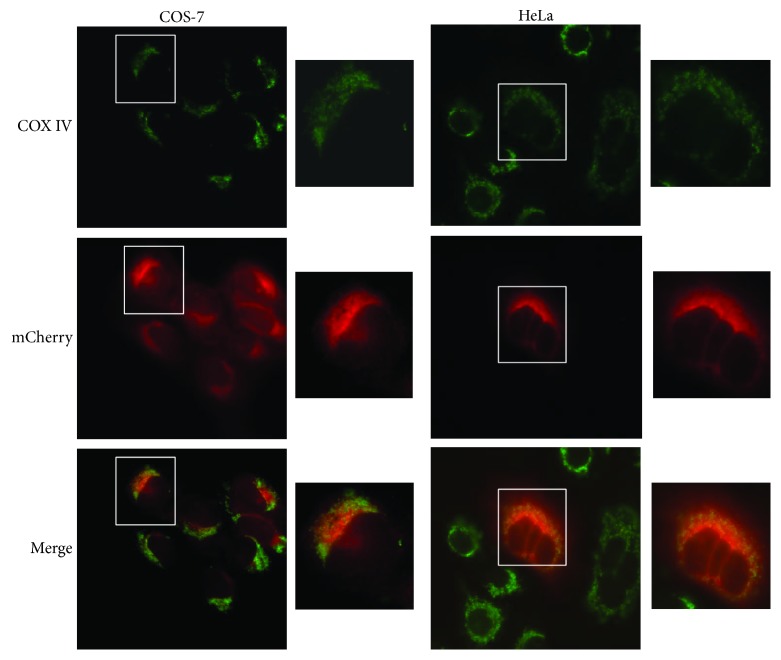
The mtmCherry colocalized the mitochondria with COX IV, ×200. COS-7 and HeLa cells were transfected with mtmCherry plasmids. At 30 h posttransfection, the cells were stained with COX IV, and COX IV (green) and mCherry (red) expressions were observed.

As shown in Figure 2, the fluorescence images clearly indicated that COX IV expressed in the mitochondria, and mCherry also specifically localized to the same sites. Hence, it demonstrated that Pink1-MTS sequence might mediate mCherry to localize mitochondria.

### 3.2. ROS Induced by mtKR Exposed to Visible Light

**Figure 3:**
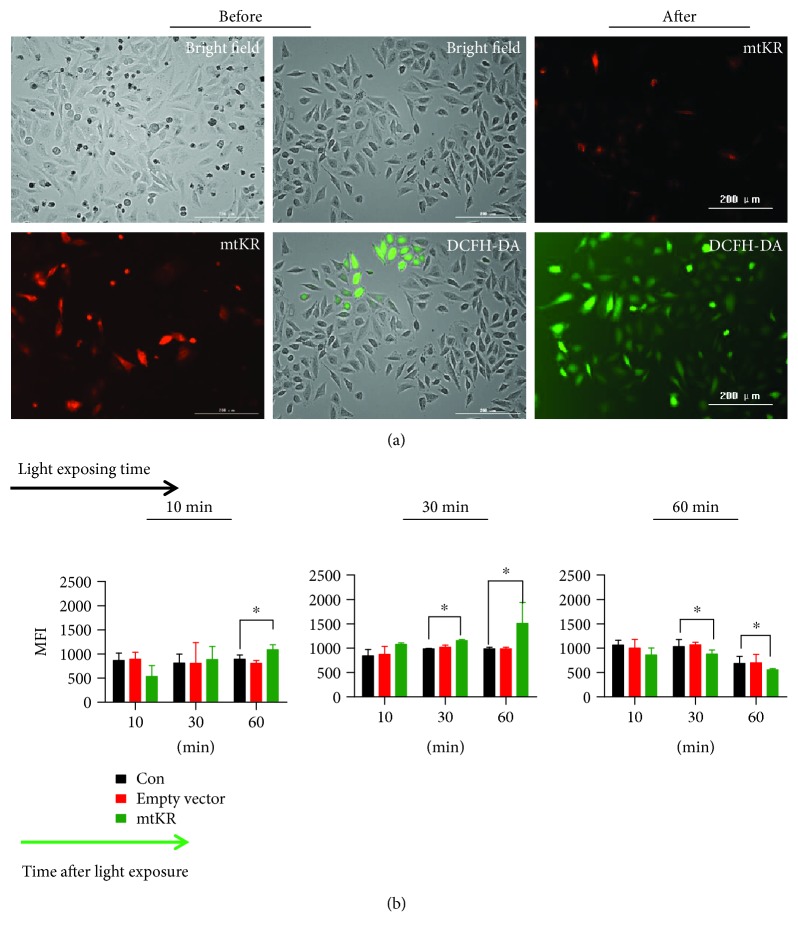
ROS changes measured by DCFH-DA staining. (a) The images of mtKR (red) before or after light exposure for 30 min in HeLa cells and cells stained by DCFH-DA (green), scale bars: 200 *μ*m. (b) The changes of MFIs at different time postlight exposure for 10, 30, and 60 min, respectively. The bars represent the mean ± SD of triplicate measurements. ^∗^*P* < 0.05 versus control.

As shown in Figure 3(a), before light exposure, there were a large amount of red cells and very few green cells; after light exposure, red cells decreased and green cells increased, indicating ROS production. As shown in Figure 3(b), at 60 min post-10 or -30 min light exposure, MFIs reached for maximum value, but at 30 or 60 min post-60 min light exposure, MFIs reduced. Taken together, these results indicated that light exposure caused the inactivation of mtKR protein and the increase of ROS.

### 3.3. Mitochondrial Dysfunction Caused by mtKR and Irradiation

**Figure 4:**
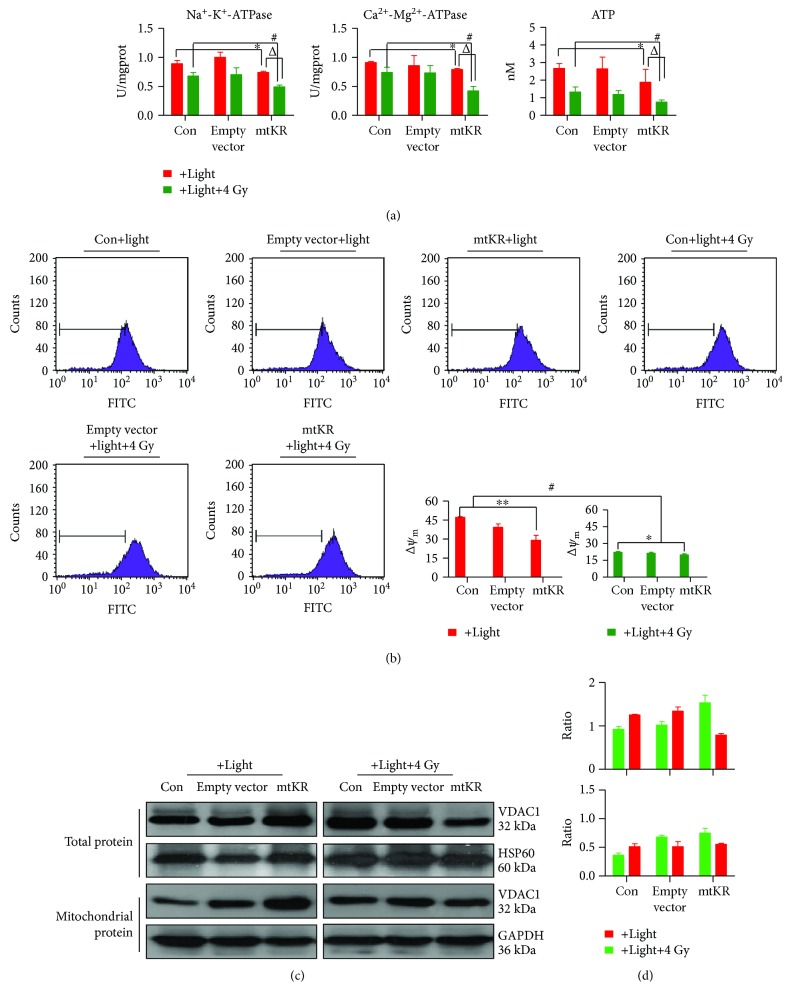
Mitochondrial dysfunctions caused by mtKR-induced ROS and X-rays. (a) The changes of Na^+^-K^+^ and Ca^2+^-Mg^2+^ ATPase activities and ATP content by biochemical assay after light exposure and irradiation. (b) The FCM pictures of Δ*ψ*_m_. HeLa cells were stained by Rh123, followed by FCM analysis of the Δ*ψ*_m_. (c) Western blot analysis was performed to determine the protein levels of VDAC1 in total and mitochondrial protein. GAPDH and HSP60 proteins were used for loading control. (d) From top to bottom, the gray ratios of VDAC1/GAPDH and VDAC1/HSP60. The bars represent the mean ± SD of triplicate measurements. ^∗^*P* < 0.05 versus control; ^#^*P* < 0.05 versus 4 Gy irradiation, and ^△^*P* < 0.05 versus light exposure.

The Na^+^-K^+^ and Ca^2+^-Mg^2+^ ATPase activities and ATP content significantly decreased after light exposure and irradiation (Figure 4(a)). Additionally, even though Δ*ψ*_m_ significantly reduced, it had similar change regularity as ATPase activities (Figure 4(b)). As shown in Figures 4(c) and 4(d), VDAC1 expressions in total and mitochondrial proteins were all increased, but after 4 Gy irradiation, VDAC1 decreased in total protein. Taken together, these results showed that mtKR-induced ROS and X-rays caused mitochondrial dysfunction, and MPTP was kept in opening status.

### 3.4. Changes of Apoptotic Rate Caused by mtKR and Irradiation via Cyt c/Caspase-3 Pathway

**Figure 5:**
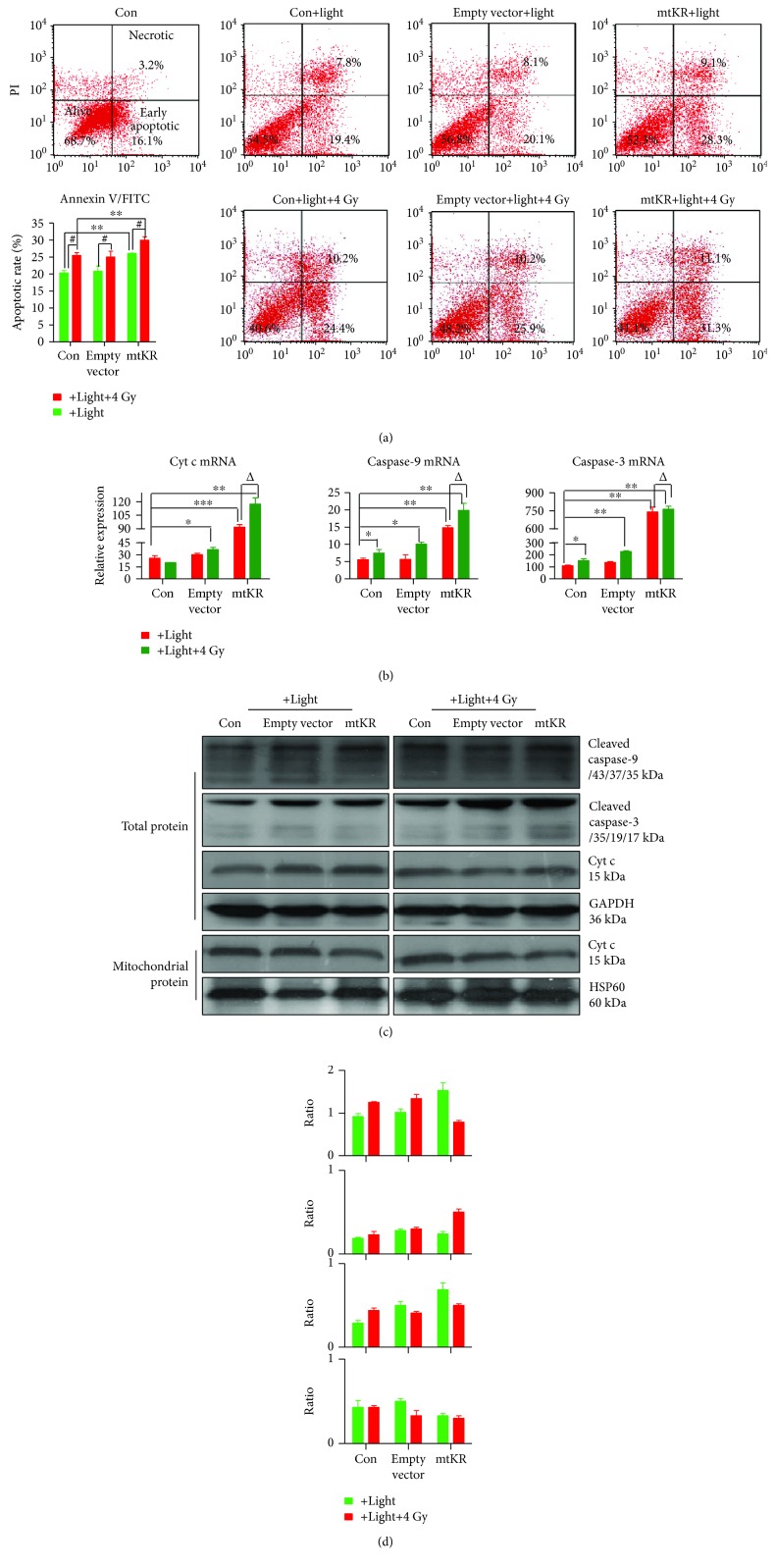
Apoptosis induced by mtKR and irradiation via Cyt c/caspase-3 pathway. (a) The FCM pictures of apoptosis and FCM analysis in HeLa cells stained by Annexin V/FITC and PI; the apoptotic population was defined as early apoptosis (lower right, green of FITC staining). (b) Cyt c, caspase-9, and caspase-3 mRNAs were detected by qRT-PCR. (c) Western blot was performed to determine the protein levels of Cyt c, caspase-9, and caspase-3 in total and mitochondrial proteins. GAPDH and HSP60 proteins were used for loading control. (d) From top to bottom, the gray ratios of cleaved caspase-9/GAPDH, cleaved caspase-3/GAPDH, Cyt c/GAPDH, and Cyt c/HSP60. The bars represent the mean ± SD of triplicate measurements. ^∗^*P* < 0.05, ^∗∗^*P* < 0.01, and ^∗∗∗^*P* < 0.001 versus control; ^#^*P* < 0.05 versus 4 Gy irradiation, and ^△^*P* < 0.05 versus light exposure.

At 12 h postirradiation, early apoptotic rates caused by mtKR exposure to light were significantly increased, and 4 Gy X-rays also induced the increase of apoptotic rate (Figure 5(a)). As shown in Figure 5(b), at 24 h postirradiation, the mRNA expressions of Cyt c, caspase-9, and caspase-3 dramatically increased. And caspase-9 and -3 proteins were cleaved into active fragments in total proteins; Cyt c protein expression reduced in mitochondrial protein; however, it increased in total protein (Figures 5(c) and 5(d)).

## 4. Discussion

Radiotherapy is the major means of cancer treatments, and its major feature is the induction of toxic oxidative damage in targeted cancer cells. Under normal physiological condition, cells maintain a basal redox balance between prooxidative and antioxidative reactions [30]. During radiation, ROS generated from water by radiation energy deposition can oxidize DNAs, proteins, and lipids and target mitochondria to cause mitochondrial dysfunction and final cell death. Moreover, the ROS has extremely short lifespan and a limited diffusion distance leading to low killing efficiency to tumor cells and unsatisfactory therapeutic effects [31–33]. In this study, based on KillerRed-induced ROS, we utilized Pink1-MTS to mediate mitochondrial localization, and our results also indicated our hypothesis. In addition, our data showed that mtKR might promote mitochondrial ROS burst. The nature of the cytotoxicity of KillerRed, a generator of ROS, therefore offers a significant opportunity to genetically investigate the mechanisms regulating cellular responses.

Mitochondrion is an ancient organelle generating approximately 90% of cellular ATP via oxidative phosphorylation [34]. Unlike normal cells, there is an abnormal redox status in cancer cells, which is unable to regulate redox homeostasis [35]. It is postulated that mitochondrial dysfunction in cancer cells would affect the relative cellular ATPase activities, ATP production, and subsequent apoptosis and migration processes. In the present study, the relative ATPase activity and ATP in HeLa cells transfected by mtKR plasmids were significantly decreased. Moreover, various evidences suggest that the mitochondrial dysfunction plays a key role in oxidative stress [36, 37], and ROS generation impairs mitochondrial electron transport chain [38]. The decline in Δ*ψ*_m_ is an earlier event in the process of cell death, and we also showed that mtKR-induced ROS can result in the loss of Δ*ψ*_m_. VDAC is the most abundant protein in the outer mitochondrial membrane, and the fact that VDAC plays a role in MTPT is undeniable, so it has long been considered to be a candidate for the outer membrane component of the MPTP [39]. Recently, it has been shown that the opening of VDAC is a regulated process, and VDAC may exhibit some degree of specificity in the mitochondrial import/export of molecules (e.g., ATP, Ca^2+^, and other ions) [40]. In some studies, VDAC1-deficient mitochondria isolated from a mutant yeast strain failed to exhibit the Bax/Bak-induced Δ*ψ*_m_ loss and Cyt c release that was observed with VDAC1-expressing control mitochondria [41]. Our results showed that VDAC1 protein expression significantly increased in total and mitochondrial proteins, indicating an opening status of the mitochondrial membrane. In addition, it is well known that radiation-induced increase in ROS causes DNA damage, cell cycle arrest, and activation of some transcription and apoptotic factors [42, 43]. Thus, the hypothesis that mtKR aggravates the mitochondrial dysfunction induced by radiation is understandable. Interestingly, our results also verify this hypothesis.

There are two major pathways in apoptosis [44]. One involves death receptors and is marked by Fas-mediated caspase-8 activation, and the other is the stress- or mitochondrial-mediated caspase-9 and -3 activation. Mitochondria are the major source of ROS production in cells, in turn, the most adversely affected organelles [45]. To better understand the mechanism that ROS leads to apoptosis, we demonstrated in this study that an acute burst of ROS in the mitochondria specifically resulted in the apoptosis, the subsequent Cyt c release and activation of caspase-9 and -3. Our results showed a promoting role on the apoptosis resulting from mtKR, which might be enhanced by radiation, and had impressive significance for tumor radiotherapy. The releases of Cyt c as well as other proteins from the mitochondria and cytosol appear to play a central role in the induction of the apoptotic cascade that ultimately leads to the programmed cell death [46]. To further explore the mechanisms, we analyzed the transcriptional levels and protein expressions of Cyt c, caspase-9, and caspase-3 at 24 h postirradiation, and their mRNA levels all increased. Interestingly, Cyt c protein expression increased in total protein however reduced in mitochondrial protein. Moreover, radiation could enhance these effects of mtKR, which indicated that Cyt c was released from the mitochondria. Under the downstream of mitochondrial apoptotic pathway, caspase-9 and -3 were activated (Figures 5(c) and 5(d)), which made us believe mtKR induced-ROS might act synergistically with radiation to induce the apoptosis via Cyt c/caspase-3 pathway.

**Figure 6:**
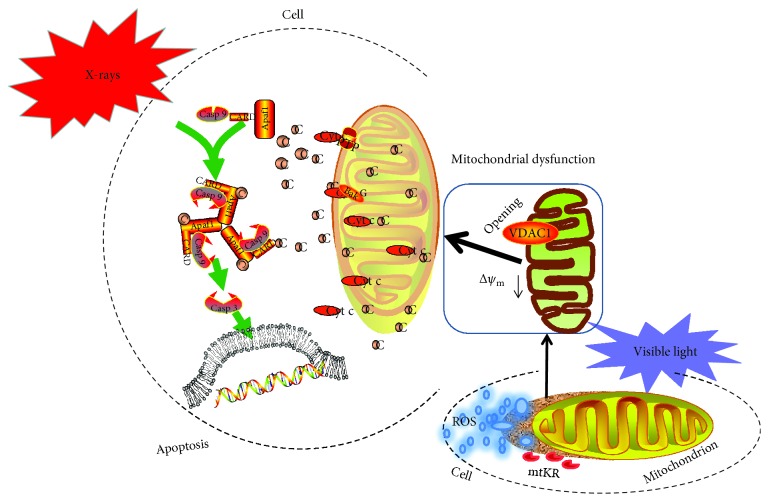
The proposed scheme of apoptosis induced by mtKR and radiation via Cyt c/caspase-3 pathway.

In conclusion, as illustrated in Figure 6, this present study demonstrates that the mitochondrial targeting characteristics of Pink-MTS and ROS increased by mtKR exposure to visible light in HeLa cells and then to impaired mitochondrial function. When ATPase activities and ATP content as well as Δ*ψ*_m_ reduced, and VDAC1 expression increased, the cell apoptosis also increased dependently on the Cyt c/caspase-3 pathway. Notably, mitochondrial dysfunction and final apoptosis enhanced by radiation has provided a new strategy for ROS sensitization in future clinical cancer therapy.

## Data Availability

In these studies, all data were obtained by PCR technique, flow cytometry (FCM), biochemical assay, observation by fluorescence microscope, quantitative real-time PCR, and Western blot, and some pictures were plotted using different tools, such as BVTech plasmid software, Adobe Photoshop CS2 software, SPSS 24.0 version, GraphPad prism 6.0 software, and PPT of Microsoft office, ScienceSlides, etc. The data (the schematic diagram of vector construction, the cell picture under fluorescence microscope, some pictures drawn with experiment results by GraphPad prism 6.0 software, FCM pictures, Western blot pictures, and the proposed scheme of conclusions) used to support the findings of this study are included within the article.
